# Amorfrutins Relieve Neuropathic Pain through the PPAR*γ*/CCL2 Axis in CCI Rats

**DOI:** 10.1155/2021/8894752

**Published:** 2021-01-22

**Authors:** Pengfei Gao, Jiayu Wang, Zhen Su, Fayin Li, Xianlong Zhang

**Affiliations:** Department of Anesthesiology, The Affiliated Huaian No. 1 People's Hospital of Nanjing Medical University, Huaian, China

## Abstract

Neuropathic pain is a public health problem. Although many pharmaceuticals are used to treat neuropathic pain, effective and safe drugs do not yet exist. In this study, we tested nociceptive responses in CCI rats, and ELISA assay was performed to examine the expression of proinflammatory cytokines. We found that amorfrutins significantly reduce the pain behaviors in CCI rats and suppress the expression of proinflammatory cytokines (TNF*α*, IL-6, and IL-1*β*) and chemokines (CCL2/CCR2) in the spinal cord. However, concurrent administration of a PPAR*γ* antagonist, GW9662, reversed the antihyperalgesic effect induced by amorfrutins. The results indicate that amorfrutins inhibit the inflammation and chemokine expression by activating PPAR*γ*, thus relieving neuropathic pain in CCI rats. Therefore, PPAR*γ*-CCL2/CCR2 pathway might represent a new treatment option for neuropathic pain.

## 1. Introduction

Neuropathic pain is maladaptive pain caused by a lesion or disease affecting the somatosensory system [1]; its clinical symptoms are spontaneous ongoing or shooting pain and stimulus-evoked pain [2]. Pharmacological treatment is the first-line options for neuropathic pain; however, the currently used drugs such as tricyclic antidepressants and serotonin-noradrenalin reuptake inhibitors have limited efficacy and serious side effects [3]. Therefore, the mechanisms of neuropathic pain should be further explored to develop novel therapeutic strategies. Current studies have shown that proinflammatory cytokines contribute to the generation and maintenance of neuropathic pain [4]. Based on this concept, it provides possibilities concerning other treatment strategies.

Peroxisome proliferator-activated receptors (PPARs) are ligand-activated transcription factor belonging to a nuclear hormone receptor superfamily, containing three isoforms PPAR*α*, PPAR*β*/*δ*, and PPAR*γ* [5]. PPAR*γ* is ubiquitously expressed throughout the body; it is the regulator of adipocyte differentiation and plays a role in lipid metabolism and glucose homeostasis. In addition, increasing researches have illustrated the pivotal roles of PPAR*γ* in inflammatory gene repression [6]. PPAR*γ* agonists could suppress the production of proinflammatory cytokines in cultured monocytes [7]. As the natural agonists of PPAR*γ*, amorfrutins are found in *Glycyrrhiza foetida* and *Amorpha fruticosa* [8]. Amorfrutins bind to and activate PPAR*γ* without these side effects caused by synthetic PPAR*γ* agonists [9].

Chemokines and their receptors are key mediators of inflammation [10]. Chemokines are induced by proinflammatory cytokines and modulate immune cell recruitment into inflamed tissues. However, elevated expression of chemokines contributes to chronic inflammation, which plays a role in neuropathic pain [11]. Experimental evidence has shown that several chemokines are linked to neuropathic pain in animal models [12], and CCL2/CCR2 signaling has garnered great attention. CCL2 belongs to CC chemokine subfamily and preferentially binds the CCR2 chemokine receptor [13, 14]. In neuropathic pain models, the development of mechanical allodynia was totally abrogated in CCR2−/− mice [15]. In other models, CCL2 and CCR2 remain upregulated for a long period after injury [16]. These researches represent a promising therapeutic target.

Neuropathic inflammation contributes to the maintenance of neuropathic pain, but PPAR*γ* can inhibit the inflammation gene expression. Chemokines play a role in neuropathic pain as key inflammatory mediators [17]. Accordingly, the aim of this study was to assess whether amorfrutins can alleviate pain through PPAR*γ*/CCL2 signaling in neuropathic pain models.

## 2. Materials and Methods

### 2.1. Cell Culture

HMC3 cell line was obtained from Procell (Wuhan, China) and cultured in Modified Eagle's Medium (MEM) supplemented with 10% fetal bovine serum (FBS) and 1% P/S under 5% CO_2_ at 37°C.

### 2.2. Animal Models

Chronic constriction injury (CCI) model was established according to procedures described by Bennett and Xie [18]. Rats were anesthetized with pentobarbital. An incision was made just below the hip bone, parallel to the sciatic nerve. The nerve was exposed, and four 4-0 hromic gut sutures were used to loosely ligate the nerve with 1 mm intervals. The same surgery was performed in the sham operation group except ligating the sciatic nerve. There is no autophilia in CCI rats. The behavioral performance of the sham operation group is the same as before the operation.

All male Sprague-Dawley rats (200-300 g) were obtained from Huai'an First People's Hospital. The rats were randomly divided into four groups: (a) the sham group treated with vehicle, (b) chronic constriction injury (CCI) rats treated with vehicle, (c) chronic constriction injury (CCI) rats treated with amorfrutins (60 mg/kg), and (d) chronic constriction injury (CCI) rats treated with amorfrutins and GW9662 (30 mg/kg).

### 2.3. Behavioral Testing

#### 2.3.1. PWMT

Paw withdrawal mechanical threshold (PWMT) was tested using the electric von Frey filament (IITC, USA). Put the rats into separate plexiglas boxes with a metal mesh floor. Before the test, the rats were adapted for 30 minutes to eliminate tension. The von Frey filament was pointed at the plantar surface of rats. When the rats show paw withdrawal reaction, the value of electric von Frey filament was considered as the paw withdrawal threshold. Each measurement should be repeated 3 times at 5 minutes interval.

#### 2.3.2. PWTL

Paw withdrawal thermal latency (PWTL) was performed using the Plantar Analgesia Meter for thermal paw (IITC, USA). The rats were adapted to the environment for more than 30 minutes. Then, slide the test head and align the heat source with the bottom of the rats' hind paw. Set the stimulation time within 30 s, and automatically record the time of rats show paw withdrawal reaction. The interval between each measurement is more than 5 minutes. Repeat the measurement 3 times and take the average.

### 2.4. ELISA

The expression of TNF-*α*, IL-1*β*, and IL-6 in the spinal cord was examined using the LEGEND MAX™ Rat TNF-*α* ELISA Kit (Biolegend, China), Rat IL-1*β* ELISA Kit (Dakewe, China), and LEGEND MAX™ Rat IL-6 ELISA Kit (Biolegend, China).

### 2.5. Western Blot

The rats in each group were immediately decapitated after completing the pain behavior test on the 14th treatment day. Take out the L4~6 spinal cord, quickly put it in liquid nitrogen, and then transfer to the -80°C refrigerator.

The frozen samples were lysed using lysis buffer containing protease inhibitors. Protein (30 *μ*g) was separated by sodium dodecyl sulphate-polyacrylamide gel electrophoresis. After the protein was transferred to polyvinylidene fluoride membranes, the membranes were incubated with primary antibodies (anti-CCL2, anti-CCR2, and anti-*β* actin; 1 : 2000; Abcam, UK) and HRP-conjugated secondary antibodies (1 : 10000; Amyjet, China). The protein bands were visualized using the Clarity Western ECL Substrate (Bio-Rad, USA).

### 2.6. Statistical Analysis

The significance between groups was analyzed by Student's *t*-test and one-way ANOVA. All statistical analysis was performed using SPSS 22.0 (IBM, Chicago, USA) and GraphPad Prism 8.0.0 (San Diego, California, USA).

## 3. Results

### 3.1. Amorfrutins Suppress CCL2/CCR2 Expression through PPAR*γ* Activation

**Figure 1:**
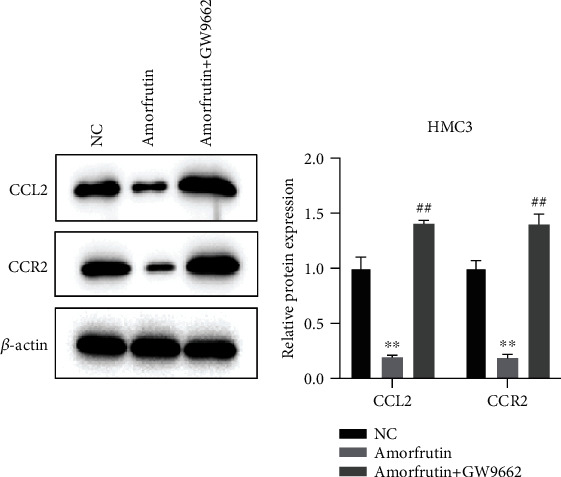
Amorfrutins suppressed the CCL2/CCR2 expression by activating PPAR*γ*. The CCL2 and CCR2 protein expressions were significantly decreased in the amorfrutin group, whereas the GW9662 reversed the effects induced by amorfrutins.

To explore the connection between PPAR and chemokines in neuropathic pain, we detected the CCL2/CCR2 expression in LPS-induced HMC3 cells. As shown in Figure 1, amorfrutins remarkably decreased the CCL2/CCR2 protein expression, whereas coadministration of amorfrutins and GW9662 (PPAR*γ* antagonist) restored the expression of CCL2/CCR2. The results indicated that amorfrutins could suppressed the expression of CCL2/CCR2 protein through PPAR*γ* activation.

### 3.2. Amorfrutins Relieve the Neuropathic Pain Responses in CCI Rats

**Figure 2:**
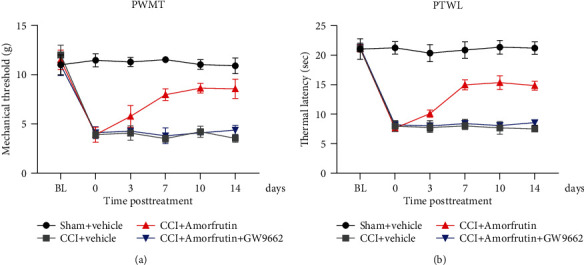
Amorfrutins relieved the neuropathic pain responses in CCI rats. (a) The PWMT of different groups were measured using the electric von Frey filament. (b) The PTWL of different groups were measured using the Plantar Analgesia Meter for thermal paw. Amorfrutins increased the PWMT and PTWL of CCI rats by activating PPAR*γ*.

Based on the results of the cell experiments, the CCI rat model was used to examine the function of amorfrutins in neuropathic pain. At 3 days after CCI surgery, all groups were treated with the corresponding drugs once a day for two weeks. At 1 h after injection, PWMT was measured by the same researcher (Figure 2(a)). In CCI rats, the mechanical thresholds were significantly reduced. However, after the administration of amorfrutins, the mechanical thresholds were increased from day 3 and reached its maximum after a week. To verify whether amorfrutins relieve neuropathic pain by activating PPAR***γ***, GW9662 was coadministrated with amorfrutins. As shown in Figure 2(a), there were no significant changes in PWMT of CCI rats treated with amorfrutins+GW9662. PTWL was performed after PWMT (Figure 2(b)). Consistent with the above results, amorfrutins remarkably alleviated the thermal allodynia of CCI rats, but GW9662 reversed this effect. The results showed that amorfrutins relieved neuropathic pain in CCI rats by activating PPAR***γ***.

### 3.3. Amorfrutins Reduce the Inflammation in CCI Rats

**Figure 3:**
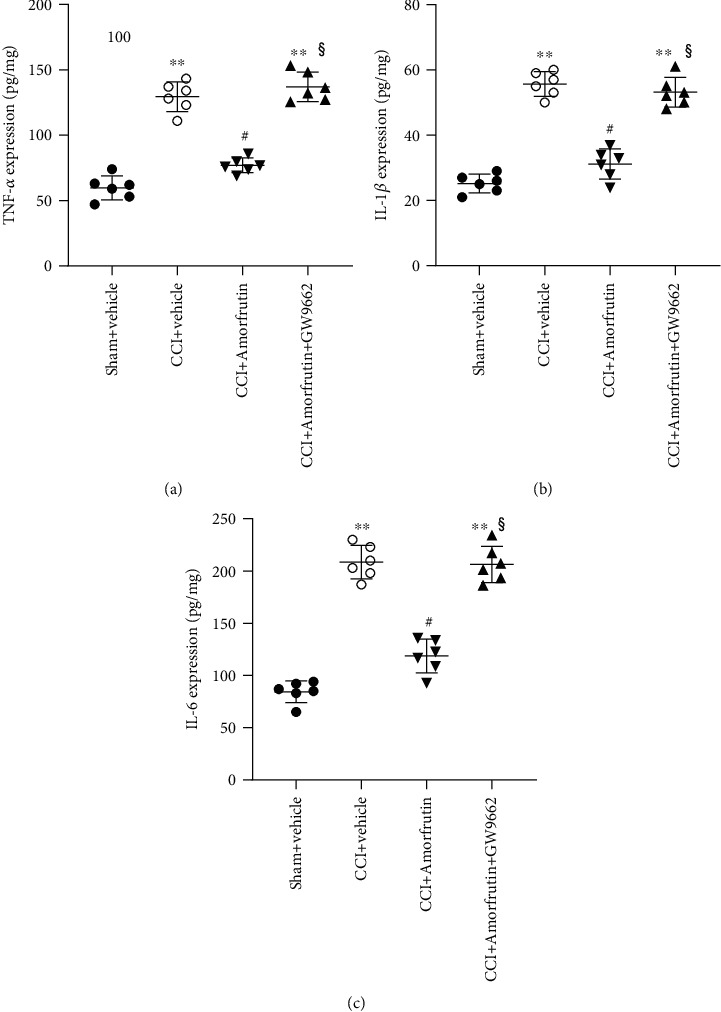
Amorfrutins reduced the inflammation in CCI rats. (a) The TNF-*α* expression in the spinal cord of different groups were detected using ELISA. (b) The IL-1*β* expression in spinal cord of different groups were detected using ELISA. (c) The IL-6 expression in spinal cord of different groups was detected using ELISA. Amorfrutins reduced the levels of TNF-*α*, IL-1*β*, and IL-6 through PPAR*γ* activation.

Inflammation has been proven to contribute to the maintenance of neuropathic pain. To verify whether amorfrutins reduce neuropathic pain is related to inflammation, the proinflammatory cytokines in the spinal cord including TNF-*α*, IL-1*β*, and IL-6 were detected using ELISA (Figures 3(a)–3(c)). The results showed that the levels of TNF-*α*, IL-1*β*, and IL-6 were higher in the CCI groups than in the sham group. And amorfrutin administration markedly inhibited the upregulation of these proinflammatory cytokines. However, GW9662 reverses the effects induced by amorfrutins, indicating that amorfrutins decrease the inflammation by activating PPAR*γ*. These findings revealed that amorfrutins might alleviate neuropathic pain by reducing inflammation.

### 3.4. PPAR*γ* Activation Suppresses Chemokines CCL2/CCR2 Expression in CCI Rats

**Figure 4:**
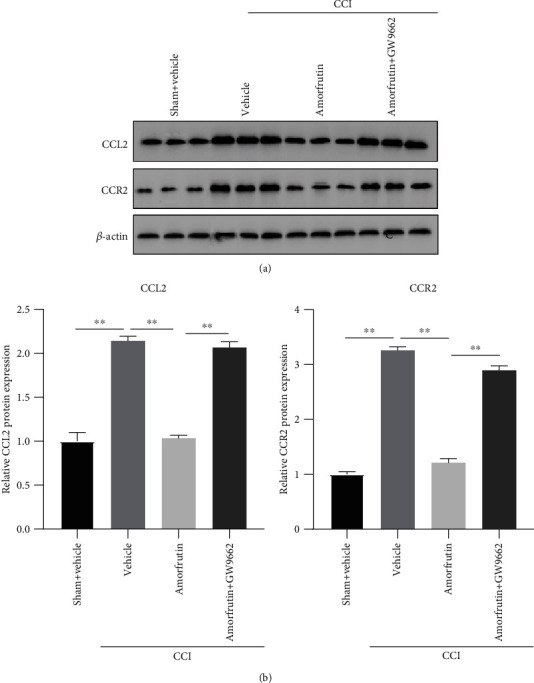
PPAR*γ* activation suppressed chemokines CCL2/CCR2 expression in CCI rats. The expression of CCL2/CCR2 protein was examined by western blot. Amorfrutins decreased the CCL2/CCR2 expression through the PPAR*γ* activation.

To further explore the connection between PPAR*γ* and chemokines in neuropathic pain, we detected the CCL2 and CCR2 expression in the spinal cord (Figure 4). Compared with the sham group, the CCL2/CCR2 expression in the CCI group was increased. In those drug-treated rats, amorfrutins significantly reduced the expression of CCL2/CCR2; however, GW9662 blocked this effect and restored the CCL2/CCR2 expression. The results indicated that amorfrutins (PPAR*γ* agonist) inhibited chemokine CCL2/CCR2 expression through PPAR*γ* activation. In summary, these data established the link between PPAR*γ* and CCL2 in neuropathic pain, which may represent a novel therapy for neuropathic pain.

## 4. Discussion

In this report, we showed that amorfrutins significantly reduce neuropathic pain in CCI rats. And the levels of inflammation cytokines and chemokines CCL2/CCR2 were decreased. In addition, PPAR*γ* antagonist GW9662 reversed the changes produced by amorfrutins.

Increasing evidence has shown that PPAR activation plays a role in alleviating neuropathic pain. In animal models, PPAR agonists pioglitazone, rosiglitazone, and palmitoylethanolamide (PEA) and fenofibrate have been proven to reduce pain [19]. In humans, the endogenous PPAR*α* agonist PEA shows great efficacy in the treatment of various human pain conditions, including diabetic neuropathy, sciatic pain, and postoperative pain [20, 21]. Little information is available on the use of PPAR*γ* agonists for neuropathic pain treatment in humans, partly because the undesirable side effects of the key agonists, thiazolidinediones (TZDs). However, amorfrutins are natural PPAR*γ* agonists, showing the anti-inflammatory effect in HFD mice without unwanted side effects [9]. In this study, our findings represent that PPAR*γ* agonist amorfrutins attenuate mechanical hyperalgesia and thermal hyperalgesia in CCI rats. PPAR*γ* antagonist GW9662 coadministration with amorfrutins blocked the role of Amor, indicating that amorfrutins alleviate neuropathic pain by activating PPAR*γ*. These data suggest that amorfrutins may be a new drug therapy for neuropathic pain.

Chemokine expression is stimulated by inflammatory cytokines like TNF*α* and IL-1*β*. Researches have revelated the connection between chemokines and pain; the chemokine expression was upregulated in animal models and maintained for weeks [22]. In addition, the connection between PPAR and chemokines was also revealed in some studies. For example, in traumatic brain injury model, the CCL2 expression was significantly suppressed by TZDs [23]. 15d-PGJ2 and rosiglitazone also inhibited the CCL2 production in LPS-stimulated microglia [24]. Here, we found that cytokine (TNF*α*, IL-6, and IL-1*β*) and chemokine (CCL2/CCR2) expressions increased in the spinal cord of CCI rats. Amorfrutins inhibited the production of these procytokines and chemokines, but GW9662 reversed the inhibitory effect of amorfrutins. The results illustrate that the PPAR*γ* activation can reduce the inflammation and suppress the chemokine CCL2/CCR2 expression.

## 5. Conclusions

Collectively, our results demonstrate that the PPAR*γ* agonist amorfruitins alleviate neuropathic pain in CCI rats, at least in part, via downregulating proinflammatory cytokines and chemokines CCL2/CCR2. This study may suggest a potential treatment option for neuropathic pain.

## Data Availability

The data that support the findings of this study are available from the corresponding author upon reasonable request.
